# Impact of Partial Body Shielding from Very High Dose Rates on Untargeted Metabolomics in Biodosimetry

**DOI:** 10.1021/acsomega.4c05688

**Published:** 2024-07-29

**Authors:** Evan L. Pannkuk, Evagelia C. Laiakis, Guy Garty, Sunil Bansal, Meth M. Jayatilake, Yuewen Tan, Brian Ponnaiya, Xuefeng Wu, Sally A. Amundson, David J. Brenner, Albert J. Fornace

**Affiliations:** † Department of Oncology, Lombardi Comprehensive Cancer Center, Georgetown University Medical Center, Washington, District of Columbia 20057, United States; ‡ Department of Biochemistry and Molecular & Cellular Biology, Georgetown University Medical Center, Washington, District of Columbia 20057, United States; § Center for Metabolomic Studies, Georgetown University, Washington, District of Columbia 20057, United States; ∥ Department of Radiation Medicine, Georgetown University Hospital, Washington, District of Columbia 20057, United States; ⊥ Radiological Research Accelerator Facility, Columbia University, Irvington, New York 10533, United States; # Center for Radiological Research, Columbia University Irving Medical Center, New York, New York 10032, United States

## Abstract

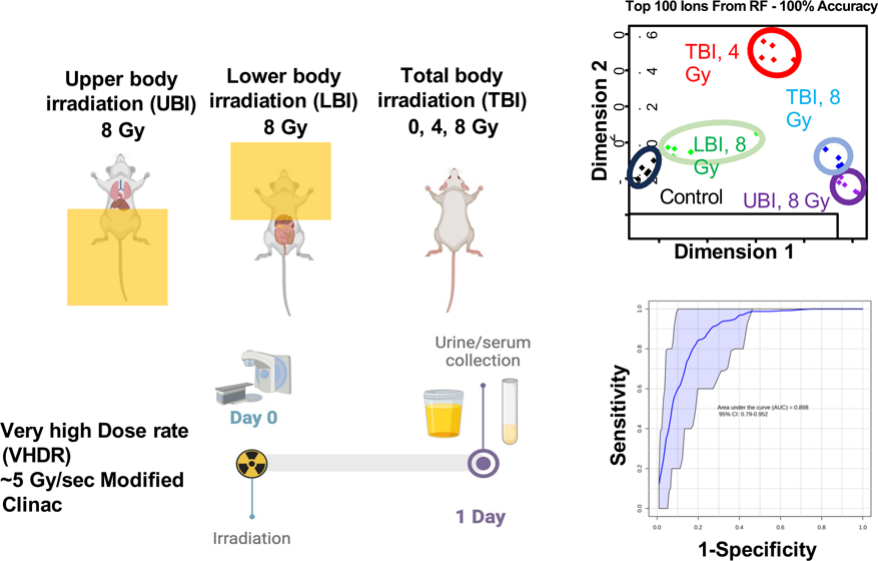

A realistic exposure to ionizing radiation (IR) from an improvised nuclear device will likely include individuals who are partially shielded from the initial blast delivered at a very high dose rate (VHDR). As different tissues have varying levels of radiosensitivity, e.g., hematopoietic vs gastrointestinal tissues, the effects of shielding on radiation biomarkers need to be addressed. Here, we explore how biofluid (urine and serum) metabolite signatures from male and female C57BL/6 mice exposed to VHDR (5–10 Gy/s) total body irradiation (TBI, 0, 4, and 8 Gy) compare to individuals exposed to partial body irradiation (PBI) (lower body irradiated [LBI] or upper body irradiated [UBI] at an 8 Gy dose) using a data-independent acquisition untargeted metabolomics approach. Although sex differences were observed in the spatial groupings of urine signatures from TBI and PBI mice, a metabolite signature (N6,N6,N6-trimethyllysine, carnitine, propionylcarnitine, hexosamine-valine-isoleucine, taurine, and creatine) previously developed from variable dose rate experiments was able to identify individuals with high sensitivity and specificity, irrespective of radiation shielding. A panel of serum metabolites composed from previous untargeted studies on nonhuman primates had excellent performance for separating irradiated cohorts; however, a multiomic approach to complement the metabolome could increase dose estimation confidence intervals. Overall, these results support the inclusion of small-molecule markers in biodosimetry assays without substantial interference from the upper or lower body shielding.

## Introduction

The potential for nuclear emergencies affecting large portions of the population exists from both terrorist threats and nuclear incidents, necessitating the continued development and refinement of radiation medical countermeasures and biodosimetry.^1^ Within the United States, the mandate for developing a research program to advance research in these areas has fallen within the Radiation and Nuclear Countermeasures Program (RNCP) of the National Institute of Allergy and Infectious Diseases (NIAID) since 2004.^2,3^ One of the primary areas highlighted includes development of radiation biomarkers for point of care, definitive dose, and predictive biodosimetry for triage and medical management for use in a nuclear emergency, such as an improvised nuclear device (IND).^4,5^ High-resolution mass spectrometry has provided an indispensable tool to this approach for its ability to rapidly quantitate metabolites (metabolomics) and proteins (proteomics), which can be damaged from free radicals produced from the indirect effects of ionizing radiation (IR) exposure and lead to altered metabolic pathways in addition to direct effects. However, experiments need to be carefully designed with respect to choosing appropriate animal models^6^ and recapitulating realistic nuclear emergency scenarios^7^ to aid biomarker discovery. Our group has developed novel irradiation systems capable of modeling variable dose rates (e.g., spanning low-dose rates from nuclear fallout to very high dose rate [VHDR] from an initial blast) and neutron exposures in murine models to elucidate how the complexity of an improvised nuclear device exposure may affect biomarker panels.^8,9^ In addition to dose rate and neutron+photon mixed exposures, different tissues have varying levels of radiosensitivity, and the effects of shielding on radiation biodosimetry need to be addressed.

Although the literature on partial body irradiation (PBI) research on more common animal models^10,11^ is too extensive to cover here, a recent series of works have comprehensively outlined the natural history of radiation injury in the Wistar rat model with ∼5% bone marrow (BM) shielding^12^ and delayed effects of radiation exposure in the C57L/J mouse model with ∼2.5% BM shielding.^13^ The overall progression of radiation injury in terms of the hematopoietic and gastrointestinal syndromes along with delayed effects was similar in these models as had been previously described, with higher acute radiation syndrome (ARS) resistance observed for C57L/J mice. In terms of metabolomics, relatively fewer studies have utilized PBI exposures, and within these, a wide range of different irradiation schemes have been used in murine models, ranging from cranial,^14^ lung^15^ (or upper body),^16^ abdominal (or lower body),^17,18^ or hepatic irradiation.^19^ Other models include NHPs^20,21^ and rats,^22,23^ with most studies measuring end points in tissues or plasma. Within studies that use PBI and are useful for biodosimetry in a nuclear disaster, end points between 1 and 7 days (d) are considered practical for definitive dose measurements. Identifying individuals requiring medical care at 1 day would be ideal, but realistically in emergency situations, it would take longer for individuals to reach testing facilities and have samples processed. At 1 day, urinary metabolites corresponding to taurine, energy (tricarboxylic [TCA] cycle intermediates), and microbial metabolism (tryptophan metabolism) have been shown to have similar changes between C57BL/6 mice exposed to total body irradiation (TBI) and PBI (thoracic, hindlimb, and abdominal), indicating their possible utility for biodosimetry.^24^ At 4 days, several serum metabolites were significantly changed in rats from control animals following abdominal radiation; however, as no comparisons were made to TBI the interpretation for biodosimetry is limited.^22^ Metabolite profiles can also be extrapolated to one to several month time points to aid in biomarkers of delayed effects of acute radiation exposure (DEARE), such as cardiac injury.^16^ As these previous studies have indicated the utility of metabolomics to span both TBI and PBI scenarios for biodosimetry, they have been confined within conventional dose rates available from commercial instruments. Further research is needed that recapitulates realistic exposure scenarios encountered during potential nuclear emergencies.

This study is an expansion of our ongoing investigations into the efficacy of biofluid metabolomic signatures across realistic exposure scenarios. As previous studies have explored the impact of neutron exposures^25−27^ and dose rate (e.g., refs (28−30) and see ref (31) for a compiled list), here we compared metabolite levels in urine and serum from male and female C57BL/6 mice following a sham irradiation, TBI of either 4 or 8 Gy, and an upper body irradiation (UBI) and lower body irradiation (LBI) with 8 Gy using a VHDR. We predicted, as we have seen in previous studies, that although certain responses to radiation injury will be specific to factors such as sex^32^ or exposure type, there will be metabolites that are universally changed irrespective of these factors. Once elucidated, these metabolites can be refined into multiplex panels to aid in high-throughput biodosimetry. This is the first study to determine biofluid metabolomics in a PBI model simulating the VHDR from realistic IND exposure.

## Materials and Methods

### Animal Models and Radiation Exposure

**Figure 1:**
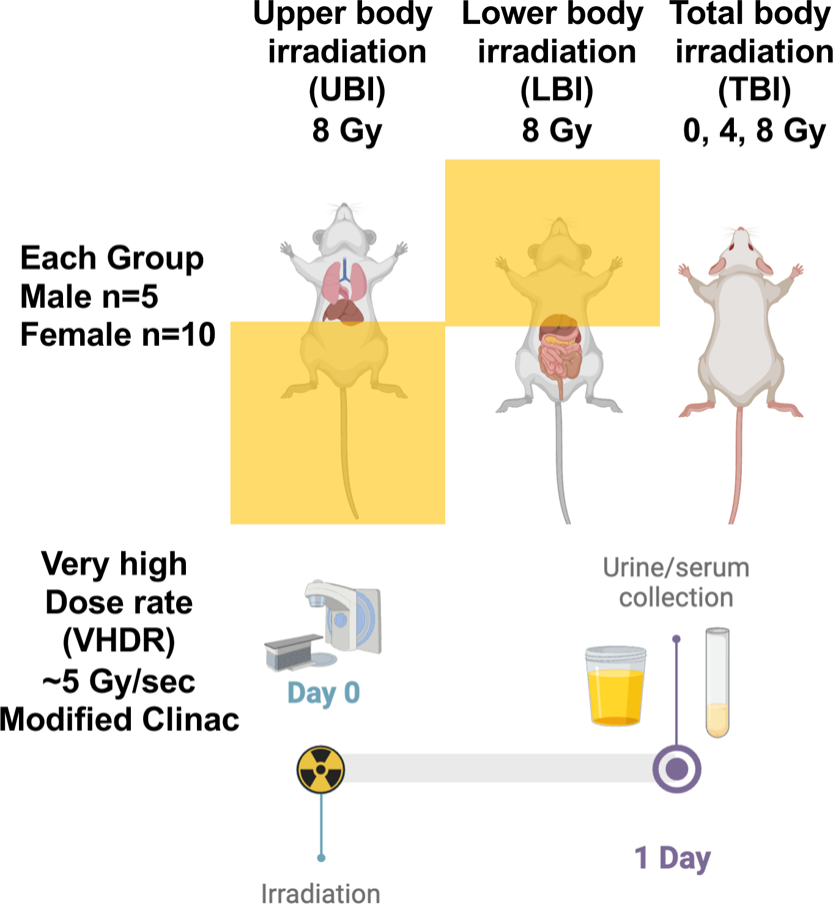
Experimental design for partial body irradiation of mice using a very high dose rate (∼5 Gy/s).

All animal experiments were approved by the Columbia University Institutional Animal Care and Use Committee (IACUC, protocol no. AABA9603) and were conducted under all relevant federal and state guidelines. Male (*n* = 5) and female (*n* = 10) C57BL/6 mice (ages 12–14 weeks) were purchased from Charles River Laboratories (Frederick, MD) and randomly assigned to the zero-dose sham (0 Gy) and irradiated (4 and 8 Gy, total and partial body exposure) cohorts (Figure 1).

All exposures were performed at the Radiological Research Accelerator Facility (RARAF), using 9 MeV electrons generated by our modified Clinac 2100C.^9^ Mice were anesthetized using isoflurane and placed into a customized irradiation jig with a movable 1/4 in.-thick lead shield allowing irradiation of the upper or lower half of the body (for PBI exposures), or no shielding for TBI. The jig was placed at a source-to-surface distance of 90 cm, and the dose was delivered at a dose rate of 5–10 Gy/s, which ensured that the circulation time of blood in the mouse, ∼15 s,^33^ was much longer than the dose delivery time (<1 s).

Dose and dose rates were evaluated prior to the experiment using a NIST-traceable advanced Markus ion chamber and Unidos E electrometer (PTW, Freiburg, Germany). EBT3 film (Ashland Specialty Chemicals, Wayne, NJ, USA) was irradiated with each mouse for dose verification and scanned using an Epson Perfection V700 scanner (Epson America, Inc., Los Alamitos, California, USA). Dose variation through the mouse thickness was previously estimated to be ±10% in this irradiation geometry.

### Chemicals

The solvents for sample preparation and LC mobile phases were Optima brand reagents from Fisher Scientific (Hanover Park, IL). Internal standards for both urine and serum were purchased from Sigma-Aldrich (St. Louis, MO) (chlorpropamide, debrisoquine sulfate, and 4-nitrobenzoic acid). Chemical standards for validations included creatine, creatinine, uric acid, l-lysine, l-arginine, l-methionine, l-tyrosine, carnitine, propionyl-L-carnitine, acetylcarnitine, Nε,Nε,Nε-trimethyllysine hydrochloride, 1-methylnicotinade chloride, 7-methylguanine, adenosine, trigonelline hydrochloride, 1-methyladenosine, uridine, citric acid, malic acid, taurine, undecanedioic acid, dodecanedioic acid, and 2-oxoadipic acid and were obtained from Sigma-Aldrich (St. Louis, MO). Xanthurenic acid was obtained from Fluka (Honeywell, Charlotte, NC). Lysophosphatidylcholine (LysoPC) (14:0), (16:0), and (18:0) were obtained from Avanti Polar Lipids, Inc. (Alabaster, AL). Hexosamine-valine-isoleucine–OH (Hex–V-I) was synthesized by Expert Synthesis Solutions (London, ON, Canada) with structure confirmation previously published.^34^ NIST plasma Standard Reference Material (SRM) 1950 (plasma) and 3667 (urine) were produced by NIST (Gaithersburg, MD).

### Untargeted Metabolite Profiling in Biofluids

Both urine and serum were prepared using a simple “dilute-and-shoot” method as we have previously described.^35^ A 20 μL aliquot of urine was mixed with 80 μL of cold 50% acetonitrile containing internal standards (2 μM debrisoquine [M + H]^+^ = 176.1188; 5 μM chlorpropamide [M + H]^+^ = 277.0414, [M-H]^−^ = 275.0257; 30 μM 4-nitrobenzoic acid [M-H]^−^ = 166.0141). The samples were vortexed and then incubated on ice for 10 min. Residual solids were pelleted to the bottom by centrifugation for 10 min (10,000*g*, 4 °C), and then, an aliquot was placed in a liquid chromatography (LC) vial. For serum, a 5 μL aliquot was mixed with 195 μL of cold 66% acetonitrile containing the same internal standards and concentrations as for urine and then prepared as above. 1 μL aliquots of each sample were combined as a quality control (QC) sample and prepared as above. Additional QC samples included the NIST Standard Reference Material 3667 (creatinine in frozen human urine) for urine and the NIST Standard Reference Material 1950 (metabolites in frozen human plasma) for serum. The QC samples were injected every 10 samples along with blanks.

Samples were injected (2 μL) into a Waters Acquity Ultra Performance Liquid Chromatography (UPLC) with a BEH C18 1.7 μm, 2.1 mm × 50 mm column and coupled to a Xevo G3 quadrupole time-of-flight (QTOF) MS (Waters, Milford, MA). We collected data in both positive and negative electrospray ionization (ESI) modes using data-independent acquisition (Lock-Spray leucine enkephalin ([M + H]^+^ = 556.2771, [M – H]^−^ = 554.2615)). For global profiling in urine samples (Figure S1), our ESI operating conditions were as follows: capillary voltage of 3.0 kV, cone voltage of 30 V, source temperature of 120 °C, desolvation temperature of 280 °C, and desolvation gas flow of 1000 L/h. Mobile phases: solvent A (water/0.1% formic acid [FA]), solvent B (acetonitrile/0.1% FA). Gradient: (solvent A and B) 4.0 min 5% B, 4.0 min 20% B, 5.1 min 95% B, and 1.9 min 5% B at a flow rate of 0.5 mL/min, column temperature 40 °C. For global profiling in serum samples (Figure S2), our ESI operating conditions were as follows: capillary voltage 2.0 kV, cone voltage 30 V, source temperature 120 °C, desolvation temperature 280 °C, and desolvation gas flow 1000 L/h. Mobile phases: solvent A (water/0.1% FA), solvent B (acetonitrile/0.1% FA), solvent C (isopropanol/0.1% FA). Gradient: (solvent A and B) 4.0 min 2% B, 4.0 min 60% B, and 1.5 min 98% B. The wash phase was 2 min 11.8% B and 88.2% C followed by reequilibration at 98% A and 2% B with a flow rate of 0.5 mL/min, column temp 60 °C.

For targeted profiling of Hex–V-I in urine samples, a 10-point standard curve was prepared (0.1–2500 ng/mL) and samples were run on a Waters Acquity UPLC with a BEH C18 1.7 μm, 2.1 mm × 50 mm column coupled to a Xevo TQ-S tandem quadrupole MS operating in multiple reaction monitoring mode (393 > 309, cone −2 eV, collision −18 eV, dwell time −0.025 s). Mobile phases: solvent D (acetonitrile [ACN]+10 mM NH_4_HCO_3_/water 95:5 v/v) and solvent E (ACN+10 mM NH_4_HCO_3_/water 1:1 v/v). Gradient: (solvent D and E) 4.0 min 95% B, 0.1 min 5% B, and 0.9 min 95% B.

### Data Processing, Statistical Analysis, and Marker Validation

For both biofluids, we manually inspected raw data files in MassLynx v.4.1 (Waters Corporation, Milford, MA) and used Progenesis QI (Nonlinear Dynamics, Newcastle, U.K.) for preprocessing, including peak alignment and picking. Adducts for compound deconvolution were set to M+H, M+2H, M+H–H_2_O, M+H-2H_2_O, M+NH_4_, M+Na (ESI^+^) or M-H, M-H_2_O–H, M+Cl, M-2H, M+FA-H (ESI−). Data was normalized to the “normalize to all compounds function” for both urine and serum. Initial identifications for spectral features were determined to ±8 ppm error of the monoisotopic mass using databases the Human Metabolome Database (HMDB)^36^ and the METLIN MS/MS empirical library.^37^ Spectral features matched to an accurate *m*/*z* (<8 ppm for the precursor ion and <20 ppm for product ions), retention time, and with a tandem MS (5–50 V ramping collision energy) fragmentation pattern matching to pure standards were assigned a metabolomics standards initiative (MSI) level 1.^38,39^ We also compared each tandem MS spectrum to the NIST/EPA/NIH Mass Spectral Library 20 v.2.4. For methyladenosine, as previously reported,^31^ we were unable to determine the specific isomer using our current method. For Hex–V-I, we used TargetLynx (Waters Corporation, Milford, MA) to determine the peak area and interpolated unknown values against a linear curve (*R*^2^ = 0.99) in GraphPad Prism 9.2.0 (GraphPad Software, La Jolla, CA). LysoPCs were identified by inspecting their MS/MS spectra and retention time to commercially available standards (LysoPC [14:0], [16:0], and [18:0]) (Figures S3 and S4).

Multidimensional scaling (MDS) plots were generated using the R v.2.15.2-based machine learning algorithm Random Forests^40^ with biofluids from the post-irradiated sham mice as the control group. Validated compounds were graphed and checked for outliers (ROUT Q = 1%), equal variances (Bartlett’s test), and normal distributions (Shapiro-Wilk test) in GraphPad Prism 9.2.0 (GraphPad Software, La Jolla, CA). Markers with significantly different variances were then compared with a Welch’s ANOVA (normal distribution) or a Brown-Forsythe test (non-normal distribution). Heatmaps were generated in MetaboAnalyst 5.0 using the ANOVA function following log transformation and Pareto scaling.^41,42^ The area under the curve (AUC) values were derived from receiver operating characteristic (ROC) curves generated in MetaboAnalyst 5.0 (Random Forests classification method).^41,42^

## Results and Discussion

### Effects of Partial Body Irradiation on Urine Markers of Radiation Injury

**Figure 2:**
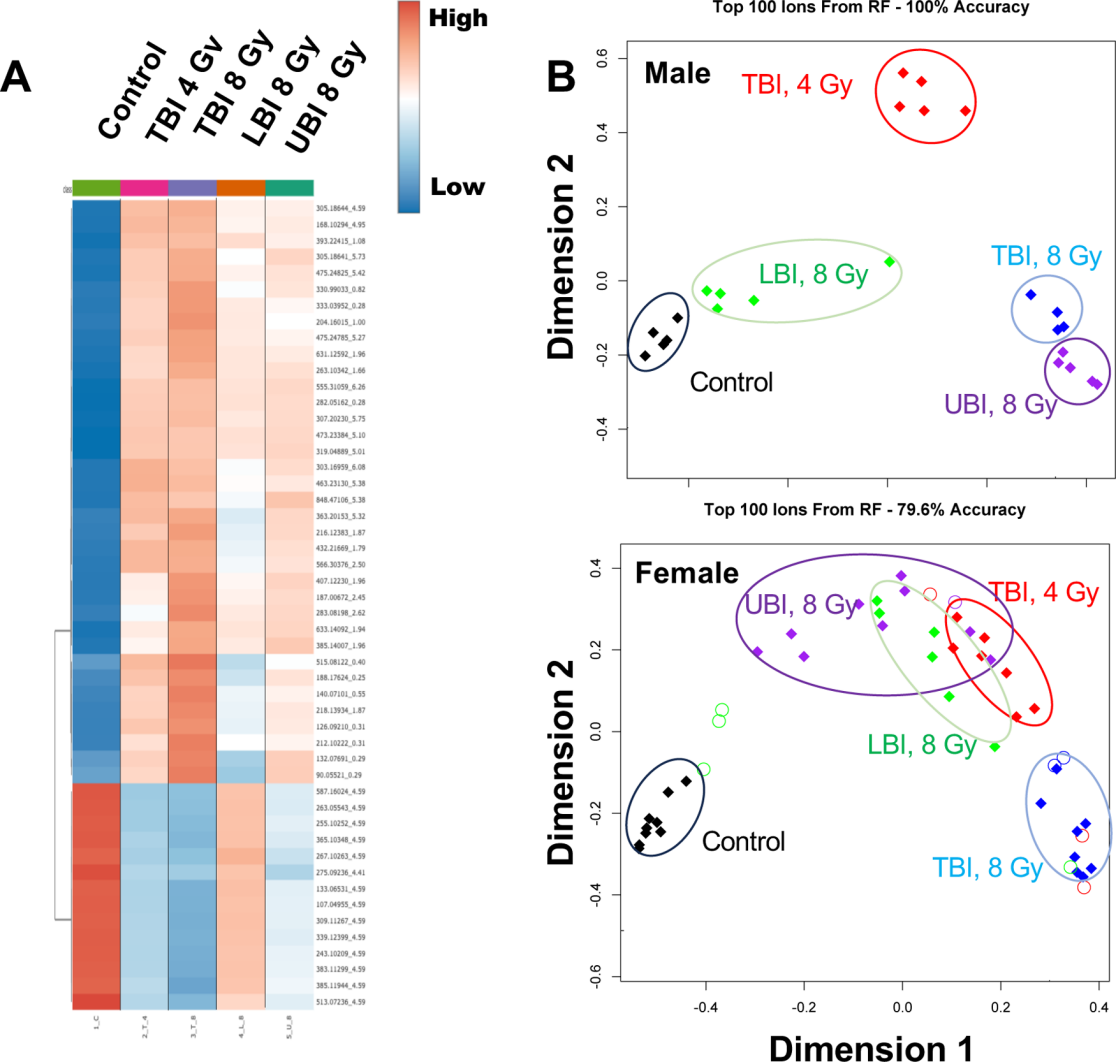
(A) Heatmap of the top 50 spectral features in ESI^+^ and ESI^–^ showing that at 1-day post-irradiation, higher perturbation is observed in the urinary metabolome following UBI compared to LBI at 8 Gy. The highest perturbation occurred following a TBI at 8 Gy followed by a TBI at 4 Gy. (B) MDS plot to visualize the combined data matrix. Different patterns are observed for males and females with high overlap between the LBI and UBI cohorts in the female mice. In males, the LBI cohort was more similar to the control group, while the UBI cohort was more similar to the TBI (8 Gy) group.

The magnitude of changes in urinary metabolites from the different irradiated cohorts is depicted as the top 50 spectral features detected in both the ESI+ and ESI– modes for urine (Figure 2A). Overall, total perturbation in the urine of PBI mice produced lower fold changes in spectral features than either TBI treatments. Higher perturbation is observed in the TBI 8 Gy cohort compared to the TBI 4 Gy cohort, which is expected. Spot urine profiles for UBI mice had increased fold changes compared to LBI, which would leave organs such as lungs, heart, and central nervous system exposed converse to upper body shielding that leaves different radiosensitive tissues exposed to IR (e.g., intestinal crypt cells and ∼60% of the bone marrow^43^). We used the Random Forests machine learning algorithm to rank the spectral features and an MDS plot to visualize the data distribution of the 4,342 spectral features detected in ESI^+^ mode and 2,845 spectral features detected in ESI^–^ mode (Figure 2B). A threshold classification accuracy of >70% was not achieved (classification accuracy = 65.8% for top 100 ions) with sexes combined, so the analysis was repeated separately for the males and females. A classification accuracy of 100% was seen in male mice, with the TBI (8 Gy) and UBI mice grouping together (Figure 2B). The LBI mice were grouped closest to the control cohort. The TBI (4 Gy) mice were grouped separately on the MDS plot for the male mice. Female mice had lower classification accuracy (79.6% for top 100 ions) compared to males and also had a differential spatial grouping. The TBI (8 Gy) mice grouped separately on the MDS plot furthest away from the control group, as expected; however, both LBI and UBI mice were not highly differentiated from the TBI (4 Gy) group. Interestingly, sex differences were not apparent at higher TBI doses (8 Gy), but PBI led to diverging signatures between males and females. Previous studies have reported differences in survival between males and females using a 2.5% bone marrow shielding model (14 Gy dose),^32^ where the males appeared more radiosensitive compared to females. In the Wistar rat model with 5% bone marrow shielding, higher mortality from ARS was observed at lower doses for males (≥8 Gy doses) compared to females (≥8.5 Gy doses), again suggesting higher radiosensitivity for males.^12^ Morbidity from DEARE at 180 days was reported to be similar for both males and females (≥8 Gy).^12^ Although sex differences were observed in these spatial modeling approaches, each significant spectral feature was analyzed to determine their potential efficacy for biodosimetry for both males and females.

**Figure 3:**
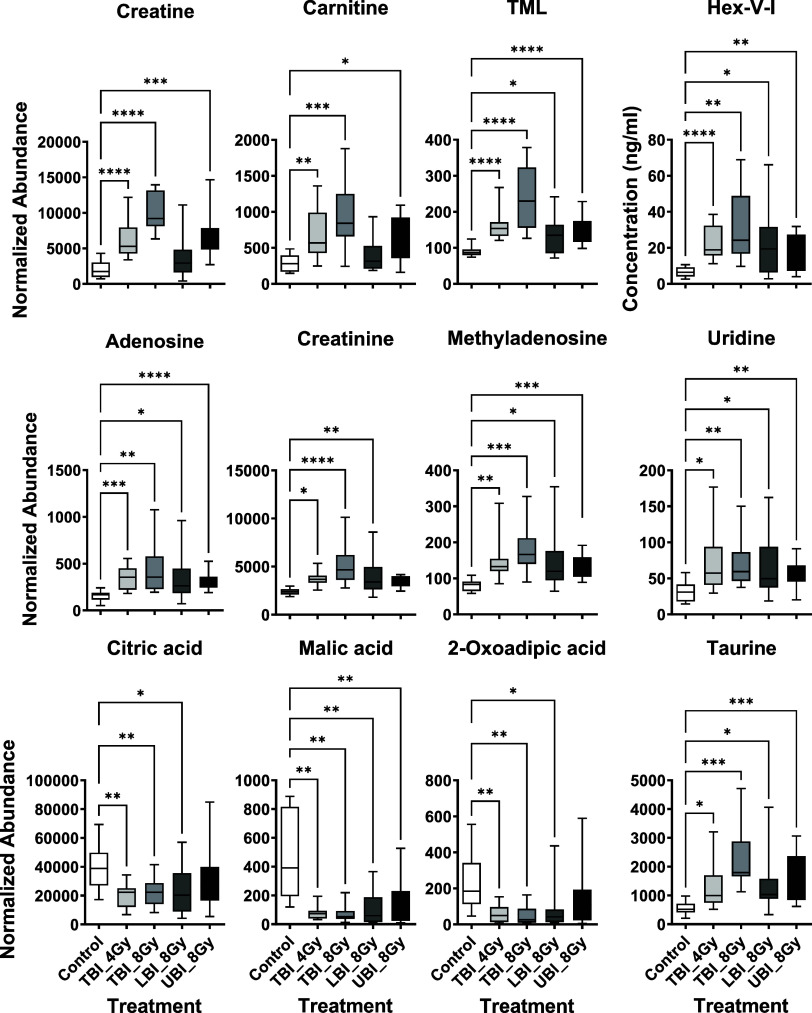
Changes in the concentration of a subset of urinary metabolites at 1 day following a TBI at either 4 or 8 Gy, an LBI at 8 Gy, or UBI at 8 Gy. (*P ≤ 0.05, **P ≤ 0.01, ***P ≤ 0.001, ****P ≤ 0.0001 false discovery rate corrected from Dunnett’s multiple comparison test. Lines from top to bottom represent max value, 75th percentile, median, 25th percentile, min value.)

Seven validated urinary metabolites were statistically significant irrespective of shielding type (Figure 3, Tables 1 and 2). Of these, adenosine has been identified in one study in C57BL/6 mouse urine after 5 Gy TBI (identified by OPLS-DA VIP groupings),^44^ but it is not a typical metabolite identified post-IR exposure. However, nucleotide metabolism intermediates generated through DNA damage were among the earliest targets discussed in urine for radiation biodosimetry assay development.^45^ Both uridine and methyladenosine were also significantly increased at 1 and 2 days post-VHDR 8 Gy exposure in a previous study examining variable dose rates on biofluid metabolite concentrations.^31^ These results support including purine and pyrimidine derivatives in biodosimetry assays; however, additional intermediates should be incorporated as the xanthine oxidoreductase system releases purine metabolites in NHP models after IR exposure (e.g., xanthine, hypoxanthine, and uric acid^46^) that are more commonly identified.^47^

**Table 1:** Validated Urine Metabolites

								MS/MS fragments		
metabolite	adduct	RT	experimental (*m*/*z*)	calculated (*m*/*z*)	mass error (ppm)	HMDB	formula	fragment 1	fragment 2	fragment 3
Urine										
creatine	H+	0.29	132.0767	132.0773	3.0	0000064	C_4_H_9_N_3_O_2_	114.0697	90.0545	87.0545
creatinine	H+	0.28	114.0662	114.0667	4.4	0000562	C_4_H_7_N_3_O	86.0736	72.0449	
carnitine	H+	0.29	162.1129	162.1130	0.6	0000062	C_7_H_16_NO_3_	103.0394	85.0305	60.0813
propionylcarnitine	H+	0.39	218.1390	218.1392	0.9	0000824	C_10_H_19_NO_4_	159.0640	144.1022	85.0287
TML	H+	0.25	189.1605	189.1603	1.1	0001325	C_9_H_20_N_2_O_2_	130.0868	84.0808	60.0816
1-methylnicotinamide	H+	0.28	137.0712	137.0714	1.5	0000699	C_7_H_9_N_2_O	94.0652	92.0493	78.0340
7-methylguanine	H+	0.37	166.0720	166.0729	5.4	0000897	C_6_H_7_N_5_O	149.0449	124.0542	94.0406
xanthurenic acid	H+	0.95	206.0448	206.0453	2.4	0000881	C_10_H_7_NO_4_	188.0347	160.0388	132.0440
adenosine	H+	0.32	268.1045	268.1046	0.4	0000050	C_10_H_13_N_5_O_4_	136.0620	119.0361	92.0255
trigonelline	H+	0.30	138.0550	138.0555	3.6	0000875	C_7_H_7_NO_2_	110.0609	94.0652	92.0496
methyladenosine	H+	0.30	282.1204	282.1202	0.7	a	C_11_H_15_N_5_O_4_	150.0774	133.0514	109.0514
uridine	H–	0.29	243.0617	243.0617	0.0	0000296	C_9_H_12_N_2_O_6_	153.0309	110.0256	82.0354
citric acid	H–	0.31	191.0194	191.0192	1.0	0000094	C_6_H_8_O_7_	111.0068	87.0070	85.0278
2-oxoadipic acid	H–	0.35	159.0297	159.0294	1.9	0000225	C_6_H_8_O_5_	115.0388	97.0346	59.0140
malic acid	H–	0.29	133.0140	133.0137	2.3	0000156	C_4_H_6_O_5_	115.0023	87.0080	71.0133
taurine	H–	0.26	124.0071	124.0068	2.4	0000251	C_2_H_7_NO_3_S	106.9797	94.9790	79.9553
Hex–V-I^b^	H+					162421477	C_17_H_32_N_2_O_8_	357.2	309.3	150.2

a Fragments for 2 methyladenosine isomers are observed.

b Hex–V-I values were determined by running in multiple reaction monitoring modes and quantifying against a 10-point std curve, PubChem CID.

**Table 2:** Fold Changes and P Values for Urine Metabolites Determined by a Welch’s ANOVA if Normally Distributed or a Brown-Forsythe ANOVA if Non-Normally Distributed

		fold change			
metabolite	*P* value	TBI 4 Gy	TBI 8 Gy	LBI 8 Gy	UBI 8 Gy
Urine					
creatine	<0.0001	3.3	5.3	1.8	3.5
creatinine	0.0002	1.7	2.2	1.7	1.5
carnitine	<0.0001	2.5	3.4	1.4	2.0
propionylcarnitine	0.0717	1.4	1.6	1.2	1.4
TML	<0.0001	1.8	2.7	1.5	1.7
Hex–V-I	0.0006	3.5	4.7	3.2	2.6
1-methylnicotinamide	0.0007	1.8	2.6	2.0	1.4
7-methylguanine	0.0039	1.4	1.7	1.6	1.3
xanthurenic acid (female)	<0.0001	1.5	1.6	1.1	1.3
xanthurenic acid (male)	0.0745	1.0	1.3	0.9	1.3
adenosine	0.0046	2.3	2.6	2.4	1.9
trigonelline	0.0221	0.9	0.9	0.9	0.9
methyladenosine	0.0008	1.8	2.2	1.8	1.6
uridine	0.0123	2.1	2.2	1.9	1.8
citric acid	0.0026	0.5	0.5	0.6	0.7
2-oxoadipic acid	<0.0001	0.2	0.2	0.4	0.6
malic acid	<0.0001	0.1	0.1	0.2	0.5
taurine	<0.0001	2.4	4.2	2.5	3.0

Both taurine and TML have been exciting urinary markers of IR exposure due to their identification in humans (TML^48^) and NHPs (taurine^49^), and we likewise observed increases in the current study irrespective of radiation shielding (Figure 3, Tables 1 and 2). Taurine is an abundant sulfur amino acid that was identified in early untargeted radiation metabolomics studies^50^ that may also confer a protective effect to radiation toxicity^15,51^ along with carnitine.^52^ TML is a methylated lysine derivative that is a precursor for carnitine synthesis, which was first proposed as a radiation marker after being identified in humans undergoing radiotherapy.^48^ Increases in mouse urinary TML levels have been identified in several experiments recapitulating realistic radiation exposures.^30,31,34^ It also increases at higher doses independent of impaired inflammatory pathways^53^ and in mice with depleted microbiomes.^54^

A previous experiment examining variable low-dose rate IR exposure replicating nuclear fallout revealed an interesting urinary spectral feature (*m*/*z* 393.2234, retention time = 1.3 min) with up to a 150-fold change in irradiated mice at early time points (<1 week).^34^ Through elucidating the tandem MS spectra, a structure consisting of a hexosamine with a valine-isoleucine dipeptide (Hex–V-I) was derived, confirmed to an MSI level 1 identification with synthesis of a chemical standard, and then validated across separate cohorts of mice.^30,31,54^ Mixed model interactions showed significant interaction effects between the microbiome, dose, and time post-irradiation for urinary levels of Hex–V-I in addition to carnitine and creatine.^54^ Although we had hypothesized Hex–V-I was associated with kidney function, it was not detected in kidney tissue following IR exposure (unpublished data) and shows relatively equal fold changes for both LBI and UBI cohorts (Figure 3, Tables 1 and 2). Hex–V-I remains a promising candidate for radiation biodosimetry as it shows among the highest fold changes post-irradiation along with creatine, carnitine, and taurine (Table 2).

Malic acid is an intermediate in the TCA cycle that is typically found at significantly reduced levels in urine along with other intermediates (e.g., citric acid or succinic acid) post-irradiation, which may indicate IR-induced mitochondrial energy dysfunction.^55−57^ These markers were previously reported to change irrespective of radiation shielding,^24^ although we found that significantly reduced levels were found after a TBI rather than PBI for both malic acid and citric acid, with very little change in citric acid following UBI (Figure 3, Tables 1 and 2). We also found reduced levels of 2-oxoadipic acid that followed a trend similar to that of citric acid. While 2-oxoadipic acid is reported less often in the literature, it has been documented in the urine of *Atm*^–*/*–^ mice^58^ and also lower in the plasma of rats following a 10 Gy abdominal exposure.^22^ It is a product of lysine metabolism via pipecolic acid metabolism, where pipecolic acid is the accumulated product typically reported after IR exposure.^58^ Its specificity to LBI may be explained by the gut microbiota being shielded in the UBI group.^59^

Both creatinine and methylnicotinamide showed higher fold changes following LBI, which would indicate a higher influence of kidney or intestinal tissue damage (Figures 3, S5, and Tables 1 and 2). The observed change in creatinine is rather straightforward, its clearance and relation to renal function was postulated in 1926,^60^ and the statistically significant changes in urine following IR exposure have been noted for its interference in data normalization. Methylnicotinamide along with other tryptophan intermediates may make promising candidates for low-dose internal uranium contamination^61^ and low-dose rate external exposure,^34^ which along with 7-methylguanine^62^ may be affected by exposure to intestinal tissues^63^ (Figure S5). An increase in 7-methylguanine has also been found in NHP urine post-irradiation.^35^

Two other common radiation metabolites, creatine and carnitine, did not change in concentration following an LBI in either males or females. Perturbation to the carnitine shuttle system after IR exposure has been reviewed.^64^ Its role in fatty acid β oxidation coupled with the known effects of IR exposure on mitochondrial dysfunction suggests that the ubiquitous changes in biofluid concentration following IR exposure are linked to energy metabolism. As high levels of creatine^65^ and carnitine^66^ are found in skeletal and cardiac muscle, defective fatty acid transport in these tissues could be a significant source for the levels observed in urine. Previous studies using murine IR-induced cardiotoxicity models showed an increased perturbation in the carnitine shuttle pathway in rat heart tissue following a 5 × 9 Gy local heart irradiation^23^ with a sex effect observed for mouse urinary carnitine levels.^16^

**Figure 4:**
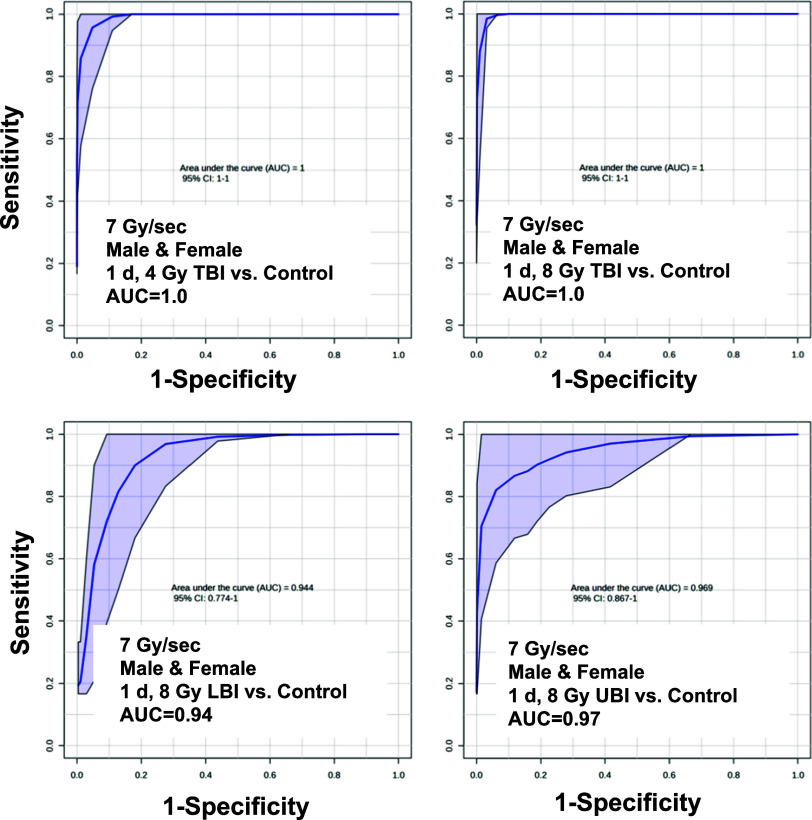
Receiver operating characteristic (ROC) curves to determine the area under the curve (AUC) for a metabolite panel in urine 1 day following a TBI at either 4 or 8 Gy, an LBI at 8 Gy, or UBI at 8 Gy. This panel (N6,N6,N6-trimethyllysine [TML], carnitine, propionylcarnitine, Hex–V-I, creatine, and taurine) was determined using a training cohort of mice that were subjected to variable dose rates simulating nuclear fallout or an initial blast. An AUC > 0.90 is considered excellent sensitivity and specificity.

Our previous study on variable dose rate examined both VHDR and fallout exposures on biofluid metabolite levels in the first 2 days of IR exposure. A urinary panel consisting of TML, carnitine, propionylcarnitine, Hex–V-I, taurine, and creatine was developed from this study,^31^ which gave excellent (AUC > 0.9) sensitivity and specificity for separating 3 and 8 Gy individuals from a nonexposed group irrespective of dose rate (Figure 4). In addition, it could identify priority individuals (8 Gy) from both zero and low exposure (3 Gy) groups combined, which could strengthen its use in the rapid screening of individuals needing immediate medical care. Here, we also achieved excellent AUC scores using the same urine metabolite panel for all radiation treatments compared to the control group (TBI 4 Gy AUC = 1.0, TBI 8 Gy AUC = 1.0, LBI 8 Gy AUC = 0.94, UBI 8 Gy AUC = 0.97) (Figure 4). These results were repeated with sexes separated, with both males and females retaining excellent AUC areas, although a slight decrease for the LBI cohort was observed for both sexes (male LBI AUC = 0.86, female LBI AUC = 0.85) (Figure S6). Also, we combined the sham-irradiated, TBI 4 Gy, LBI 8 Gy, and UBI 8 Gy groups and compared them to the TBI 8 Gy group and achieved excellent sensitivity and specificity (Figure S7). This represents individuals that may be in closer proximity to the initial blast but would have significant radiation injuries to multiple tissues compared to individuals that do not need medical care (0 Gy dose) or have some shielding as protection (PBI 8 Gy). As trigonelline was not repeatable in this study^30^ and xanthurenic acid was restricted to female mice, combined with their dietary and microbial associations, these may not be prime candidates for biodosimetry panels. However, having such a large metabolite repertoire^67^ that shows high sensitivity and specificity highlights the potential of urine as an easily accessible biofluid for radiation biodosimetry.

### Effects of Partial Body Irradiation on Serum Markers of Radiation Injury

**Figure 5:**
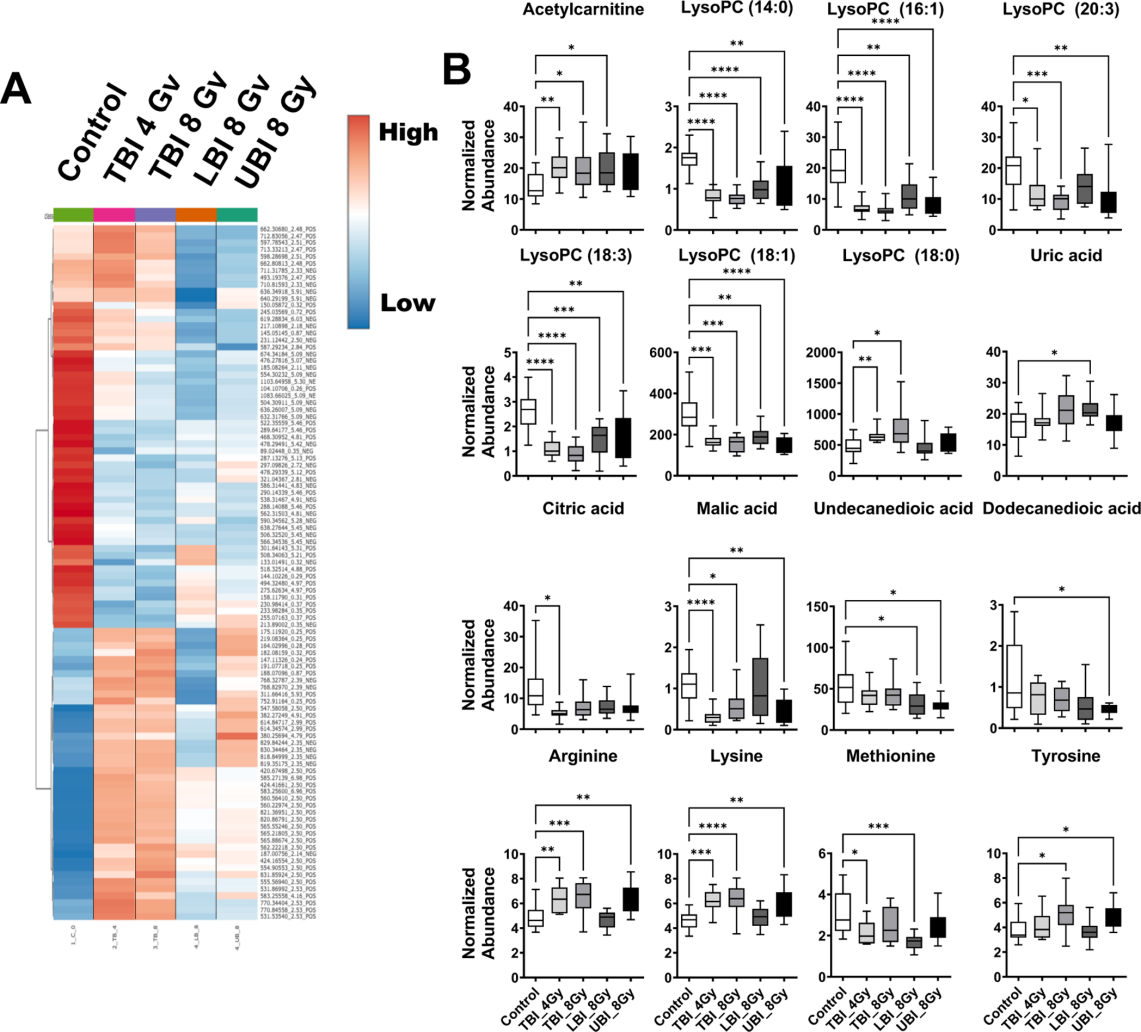
(A) Heatmap of the top 50 spectral features from serum in ESI^+^ and ESI^–^. Similar to urine, UBI and LBI showed less perturbation than the TBI groups. However, the TBI 4 and 8 Gy cohorts were more similar to what was observed in urine. (B) Changes in the concentration of a subset of serum metabolites at 1 day following a TBI at either 4 or 8 Gy, or an LBI or UBI at 8 Gy. (*P ≤ 0.05, **P ≤ 0.01, ***P ≤ 0.001, ****P ≤ 0.0001 false discovery rate corrected from Dunnett’s multiple comparison test. Lines from top to bottom represent max value, 75th percentile, median, 25th percentile, min value.)

Compared with urine, changes in serum are characterized as lower in magnitude compared to the control group following IR exposure and have a more distinct grouping between TBI and PBI mice for both males (classification accuracy = 79.2% for the top 100 ions) and females (classification accuracy = 76.0% for the top 100 ions) (Figures 5 and S8). Although both sexes exposed to TBI (both 4 and 8 Gy) and PBI (both LBI and UBI) grouped together, all irradiated male individuals were distinctly different from the control group, while females exposed to PBI exhibited a wider spread closer to the control group coupled with a higher percentage of misclassified individuals (Figure S8).

Our validated serum metabolite panel for the current TBI cohorts (16 metabolite panel) was higher than what we established for our previous VHDR studies (3 metabolite panel) (Tables 3 and 4).^30,31^ The number of metabolites and their corresponding metabolic pathways are in line with other studies following IR exposure in mouse^54^ and NHP models,^68^ where we primarily observed changes in amino acids, energy metabolites, and lipids. Of these, lipid concentrations tended to change irrespective of shielding or total body exposure (LysoPC [14:0], [16:1], [18:1], and 18:3). We have briefly reviewed serum LysoPC perturbation post-IR,^53^ which led us to follow up with a more comprehensive targeted lipidomics profiling in the serum of mice following a VHDR.^30^ More broadly, dysregulated glycerophosphatidylcholine (PC) metabolism has been of interest in cancer pathways^69,70^ and has been identified in patients undergoing radiotherapy.^71^ As a primary component of cellular membranes, PC metabolites from oxidation^72^ or enzymatic action to form lipid mediators^73^ can be abundant following physiological stressors. As with altered purine-pyrimidine metabolism, a metabolite panel for biodosimetry may contain several lipid mediators that serve as an indicator of a broader metabolic perturbation.

**Table 3:** Validated Serum Metabolites

								MS/MS fragments		
metabolite	adduct	RT	experimental (*m*/*z*)	calculated (*m*/*z*)	mass error (ppm)	HMDB	formula	fragment 1	fragment 2	fragment 3
Serum										
lysine	H+	0.24	147.1133	147.1134	0.7	0000182	C_6_H_14_N_2_O_2_	84.0810	67.0557	56.0514
arginine	H+	0.25	175.1192	175.1195	1.7	0000517	C_6_H_14_N_4_O_2_	158.0937	130.0986	70.0656
methionine	H+	0.32	150.0587	150.0589	1.3	0000696	C_5_H_11_NO_2_S	133.0323	104.0528	56.0509
tyrosine	H+	0.32	182.0816	182.0817	0.5	0000158	C_9_H_11_NO_3_	136.0759	119.0495	91.0544
acetylcarnitine	H+	0.29	204.1232	204.1236	2.0	0000201	C_21_H_17_NO_4_	145.0495	85.0282	60.0817
LysoPC (14:0)	H+	4.81	468.3095	468.3090	1.2	0010379	C_22_H_46_NO_7_P	450.2981^a^	184.0739	104.1075^b^
LysoPC (16:1)	H+	4.97	494.3248	494.3247	0.2	0010383	C_24_H_48_NO_7_P	476.3143^a^	184.0732	104.1068^b^
LysoPC (20:3)	H+	5.31	546.3558	546.3560	0.4	0010393	C_28_H_52_NO_7_P	528.3451^a^	184.0734	104.1070^b^
LysoPC (18:3)	H+	4.88	518.3251	518.3247	0.8	0010387	C_26_H_48_NO_7_P	500.3160^a^	184.0734	104.1069^b^
LysoPC (18:1)	H+	5.46	522.3556	522.3560	0.8	0010385	C_26_H_52_NO_7_P	504.3452^a^	184.0733	104.1068^b^
LysoPC (18:0)	H+	5.93	524.3712	524.3716	0.8	0010384	C_26_H_54_NO_7_P	506.3610^a^	184.0733	104.1069^b^
undecanedioic acid	H–	3.34	215.1290	215.1283	3.3	0000888	C_11_H_20_O_4_	197.1176	153.1280	57.0360
dodecanedioic acid	H–	3.63	229.1452	229.1440	5.2	0000623	C_12_H_22_O_4_	211.1329	167.1435	57.0355
citric acid	H–	0.35	191.0197	191.0192	2.6	0000094	C_6_H_8_O_7_	111.0086	87.0071	85.0308
malic acid	H–	0.32	133.0135	133.0137	1.5	0000156	C_4_H_6_O_5_	115.0023	87.0073	71.0129
uric acid	H–	0.31	167.0202	167.0205	1.8	0000289	C_5_H_4_N_4_O_3_	124.0144	96.0197	69.0091

a -H_2_O adduct

b Fragment for sn-1 isomer, stereochemistry, and double bond position cannot be determined using this method

**Table 4:** Fold Changes and *P* Values for Serum Metabolites Determined by a Welch’s ANOVA if Normally Distributed or a Brown-Forsythe ANOVA if Non-Normally Distributed

		fold change			
metabolite	*P* value	TBI 4 Gy	TBI 8 Gy	LBI 8 Gy	UBI 8 Gy
Serum					
lysine	<0.0001	1.3	1.4	1.0	1.2
arginine	<0.0001	1.3	1.3	0.9	1.3
methionine	<0.0001	0.7	0.8	0.5	0.8
tyrosine	0.0011	1.2	1.4	1.0	1.3
acetylcarnitine	0.0023	1.5	1.4	1.4	1.3
LysoPC (14:0)	<0.0001	0.5	0.5	0.6	0.6
LysoPC (16:1)	<0.0001	0.3	0.4	0.5	0.4
LysoPC (20:3)	0.0001	0.5	0.5	0.6	0.6
LysoPC (18:3)	<0.0001	0.4	0.4	0.5	0.5
LysoPC (18:1)	<0.0001	0.3	0.3	0.3	0.3
LysoPC (18:0)	0.0007	1.1	1.3	0.8	1.4
undecanedioic acid	0.0031	0.7	0.8	0.6	0.6
dodecanedioic acid	0.0025	0.6	0.7	0.4	0.5
citric acid	0.0009	0.4	0.5	0.5	0.5
malic acid	<0.0001	0.3	0.5	1.0	0.6
uric acid	0.0099	1.1	1.3	1.3	1.2

Several studies have indicated changes in amino acid levels in tissues,^18,21^ blood,^74−76^ urine,^77,78^ and nonmammalian systems^79,80^ following radiation exposure. Generation of ROS can lead to protein oxidation^81,82^ that has led to the investigation of amino acid mixtures as IR mitigators.^83^ They may also have specificity to ARS subtype, as citrulline is a specific marker of enteric dysfunction and indicates gastrointestinal syndrome.^84,85^ In this study, we found four amino acids (arginine, lysine, methionine, and tyrosine) that were statistically different in the irradiated groups compared to the control group. The relative distributions of these amino acids in the irradiated group are similar to each other, i.e., the lowest concentration is observed in the LBI group. However, basal methionine levels in the sham-irradiated mice were elevated compared to their respective irradiated groups, which complicates interpretation. As serum levels of arginine had been identified in our previous NHP studies at 1 and 7 days,^74,86^ and because of its relative abundance in serum,^87^ this was further evaluated by ROC analysis.

We found PBI-specific markers in serum, which were not observed in urine profiles. Of these, there was a decrease in long-chain dicarboxylic acids (undecanedioic acid [C11] and dodecanedioic acid [C12]), primarily associated with the UBI cohort, and an LBI-specific increase in uric acid. Changes in urinary dicarboxylic acid levels in rats following 3 Gy TBI exposure have been previously addressed in some detail.^57^ To determine dietary influence, a starvation experiment was performed in tandem with irradiation, but it was concluded that caloric restriction did not account for the full reduction in dicarboxylic acid levels observed following irradiation.^57^ Although not entirely unequivocal, the changes in both dicarboxylic acid levels and uric acid^88^ have been postulated to be associated with renal dysfunction. However, the response of urinary creatinine in the current experiment (Figure 3), a well-established biomarker of renal function, follows a clear response of what would be expected of a metabolite concentration if reduced kidney filtration was the culprit. As these results indicate similar responses for both LBI and UBI groups, the physiological mechanisms underlying long-chain dicarboxylic acid changes may require additional research.

**Figure 6:**
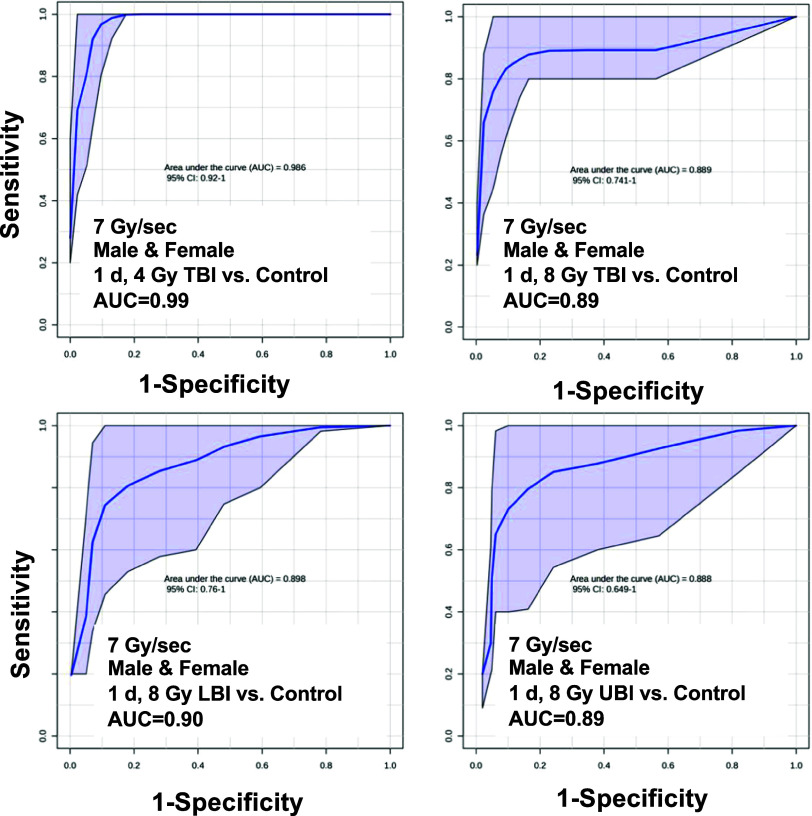
Receiver operating characteristic (ROC) curves to determine the area under the curve (AUC) for a metabolite panel in serum at 1 day following a TBI at either 4 or 8 Gy, an LBI at 8 Gy, or a UBI at 8 Gy. Several metabolites in this panel (citric acid,^54,89^ acetylcarnitine,^86^ LysoPC [14:0],^86^ [18:3], [16:1], and arginine^86^) were determined from previous nonhuman primate studies.

As few serum metabolites were identified in our previous variable dose rate study (carnitine, taurine, isobutyryl/butyrylcarnitine), we combined additional markers identified from our previous NHP studies to compose the training set. NHP models serve as the most relevant animal model to humans and several of our previous studies on post-IR effects on serum metabolite levels show altered energy metabolite and amino acid levels^55,86,89^ and lipid^90,91^ concentrations; however, using them as a model organism is prohibitive from a cost and availability standpoint. Using a combined serum panel consisting of citric acid, arginine, acetylcarnitine, LysoPC (14:0), LysoPC (18:3), and LysoPC (16:1), we achieved an excellent (AUC > 0.9) sensitivity and specificity for separating 4 and 8 Gy individuals from a nonexposed group irrespective of shielding (TBI 4 Gy AUC = 0.99, TBI 8 Gy AUC = 0.89, LBI 8 Gy AUC = 0.90, and UBI 8 Gy AUC = 0.89) (Figure 6). Analyzing males and females separately also gave good (AUC > 0.7) to excellent (AUC > 0.9) sensitivity and specificity (male: TBI 4 Gy AUC = 1.0, TBI 8 Gy AUC = 0.83, LBI 8 Gy AUC = 0.99, UBI 8 Gy AUC = 1.0; female: TBI 4 Gy AUC = 0.99, TBI 8 Gy AUC = 0.98, LBI 8 Gy AUC = 0.93, UBI 8 Gy AUC = 0.77). However, the lack of statistical significance of some metabolites in this study (carnitine, taurine, isobutyryl/butyrylcarnitine) in serum that were previously identified also highlights the need to integrate several biomarkers into a single targeted method, which is an ongoing avenue of research in our laboratory. Additionally, coelution and similar fragmentation of metabolites can lead to lack of specificity (e.g., citrulline and arginine^92^), which can be ameliorated by custom-designed targeted MS assays.

## Conclusions

High-throughput biodosimetry tests and radiation countermeasures are needed at multiple levels from rapid point-of-care devices that may be used in the field to highly automated laboratory-based approaches to screen thousands of individuals in the first week following a nuclear emergency.^5,93^ As radiation exposure affects multiple metabolic pathways, high-resolution MS has become an indispensable tool in the field of radiation biomarker research, as it can rapidly identify proteomic and metabolomic perturbations. However, radiological injury from exposure to an IND is a multifaceted scenario that may include variable dose rates and neutron + photon mixed exposures, in addition to partial shielding and other injuries, such as trauma. We previously designed novel irradiation systems that could utilize a murine model to recapitulate complex radiological exposures to test our MS-based assays. We found that urinary metabolites (TML, carnitine, propionylcarnitine, Hex–V-I, taurine, and creatine) commonly perturbed from radiation exposure could be combined to identify irradiated individuals at 1–2 days post-irradiation from VHDR and nuclear fallout-type exposures. Here, these results were repeated for cohorts of mice that were subjected to varying PBI from VHDR exposure, strengthening the broad applicability of metabolite signatures for biodosimetry. For serum metabolites, we composed a panel of metabolites that have been identified in our previous untargeted studies on NHPs that could distinguish individuals irrespective of radiation shielding; however, dose estimations based on blood may require a multiomic approach to complement the metabolome or further refining. The identification of these more universal markers is promising for the continued refinement of a field-deployable biodosimetry device.

## Data Availability

The data used in this paper can be downloaded from Metabolomics Workbench site: https://www.metabolomicsworkbench.org/

## Supporting Information Available

Base peak chromatograms in both ESI+ and ESI– for the pooled quality control urine (Figure S1); base peak chromatograms in both ESI+ and ESI– for the pooled quality control serum (Figure S2); tandem MS spectra (5–50 V ramping collision energy) of LysoPCs (Figure S3); tandem MS spectra (5–50 V ramping collision energy) of LysoPCs (Figure S4); urinary markers that were significantly perturbed following IR exposure in the current study (Figure S5); receiver operating characteristic (ROC) curves with the area under the curve (AUC) for a metabolite panel at 1 day following a TBI at either 4 or 8 Gy, an LBI at 8 Gy, or UBI at 8 Gy (Figure S6); receiver operating characteristic (ROC) curves with the area under the curve (AUC) for a metabolite panel at 1 day following a TBI at 8 Gy compared to the combined sham irradiation, TBI at 4 Gy, LBI at 8 Gy, and UBI at 8 Gy groups (Figure S7); and multidimensional scaling (MDS) plots to visualize the combined data matrix (Figure S8) (PDF)
