# Supplementary material for: Impact of Partial Body Shielding from Very High Dose Rates on Untargeted Metabolomics in Biodosimetry
Source: ACS Omega. 2024 Jul 29;9(32):35182–96. doi: 10.1021/acsomega.4c05688 (PMC11325421; doi:10.1021/acsomega.4c05688)
Supplement: Supplementary file 1 — ao4c05688_si_001.pdf [file ao4c05688_si_001.pdf]

Supporting Information

Impact of partial body shielding from very high dose rates on untargeted metabolomics in biodosimetry

Evan L. Pannkuk [1,2,3*], Evagelia C. Laiakis [1,2,3,4], Guy Garty [5,6], Sunil Bansal [1], Meth M. Jayatilake [1], Yuewen Tan [5], Brian Ponnaiya [6], Xuefeng Wu [6], Sally A. Amundson [6], David J. Brenner [6], Albert J. Fornace Jr. [1,2,3,4]

[1]Department of Oncology, Lombardi Comprehensive Cancer Center, Georgetown University Medical Center, Washington, DC 20057, United States of America
[2]Department of Biochemistry and Molecular & Cellular Biology, Georgetown University Medical Center, Washington, DC 20057, United States of America
[3]Center for Metabolomic Studies, Georgetown University, Washington, DC 20057, United States of America
[4]Department of Radiation Medicine, Georgetown University Hospital, Washington, DC 20057, United States of America
[5]Radiological Research Accelerator Facility, Columbia University, Irvington, NY, United States of America
[6]Center for Radiological Research, Columbia University Irving Medical Center, New York, NY, United States of America

Corresponding Author

*Evan L. Pannkuk, PhD
Georgetown University, 3970 Reservoir Road, NW, New Research Building, Room E504, Washington, DC, USA, 20057
E-mail: elp44@georgetown.edu, Phone: (202) 687-5650

Supplemental Figure 1. Base peak chromatograms in both ESI+ and ESI- for the pooled quality control urine sample compared to NIST SRM 3667.

Supplemental Figure 2. Base peak chromatograms in both ESI+ and ESI- for the pooled quality control serum sample compared to NIST SRM 1950 (an insert magnifies the TIC from 0 to 8.0 min).

Supplemental Figure 3. Tandem MS spectra (5 – 50 V ramping collision energy) of LysoPC (14:0), LysoPC (16:1), and LysoPC (20:3).

Supplemental Figure 4. Tandem MS spectra (5 – 50 V ramping collision energy) of LysoPC (18:0), LysoPC (18:1), and LysoPC (18:3).

Supplemental Figure 5. Additional urinary markers that were significantly perturbed following IR exposure in the current study. Both methylnicotinamide and 7-methylguanine are promising candidates for biomarker development. However, LPC

(22:6) is difficult to detect in urine, trigonelline is not repeatable and shows very low fold changes, and xanthurenic acid is specific to females.

Supplemental Figure 6. Receiver operating characteristic (ROC) curves with the area under the curve (AUC) for a metabolite panel at 1 day following a TBI at either 4 or 8 Gy, a LBI at 8 Gy, or UBI at 8 Gy. The panel (N6,N6,N6-trimethyllysine [TML], carnitine, propionylcarnitine, Hex-V-I, creatine, and taurine) with males (above) and females (below) separated out shows that this panel gives excellent (AUC > 0.9) to very good (AUC > 0.8) sensitivity and specificity for partial body shielding and sex.

Supplemental Figure 7. Receiver operating characteristic (ROC) curves with the area under the curve (AUC) for a metabolite panel at 1 day following a TBI at 8 Gy compared to the combined sham irradiation, TBI at 4 Gy, LBI at 8 Gy, and UBI at 8 Gy groups. The panel (N6,N6,N6-trimethyllysine [TML], carnitine, propionylcarnitine, Hex-V-I, creatine, and taurine) with males (above) and females (below) separated out shows that this panel gives excellent (AUC > 0.9) sensitivity and specificity for individuals receiving higher dose TBI needing immediate medical attention compared to individuals that do not need medical care (sham) or different treatment (significant bone marrow shielding).

Supplemental Figure 8. Multidimensional scaling (MDS) plots were generated to visualize the combined data matrix. Compared to urine, there is a more distinct grouping between TBI and PBI groups for both males and females; however, the male PBI individuals grouped away from the control group more distinctly than females.

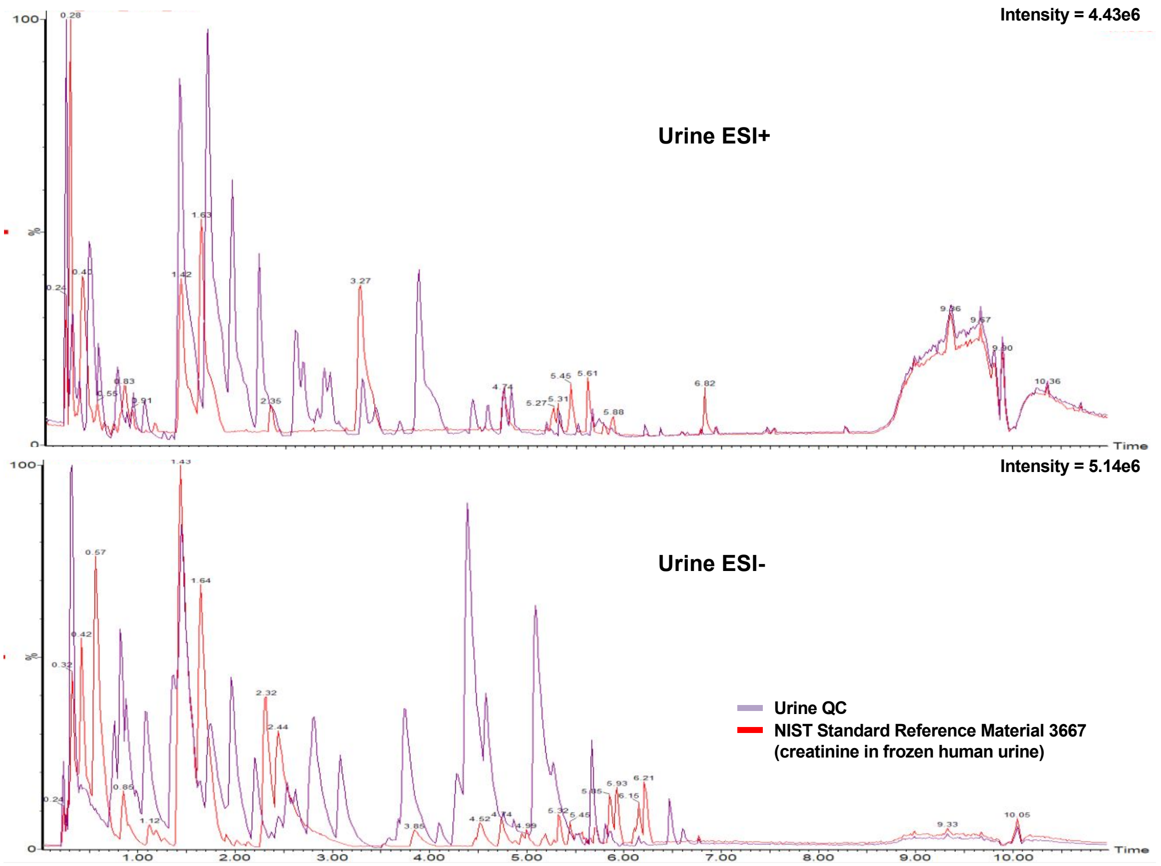

Supplemental Figure 1. Base peak chromatograms in both ESI+ and ESI- for the pooled quality control urine sample compared to NIST SRM 3667.

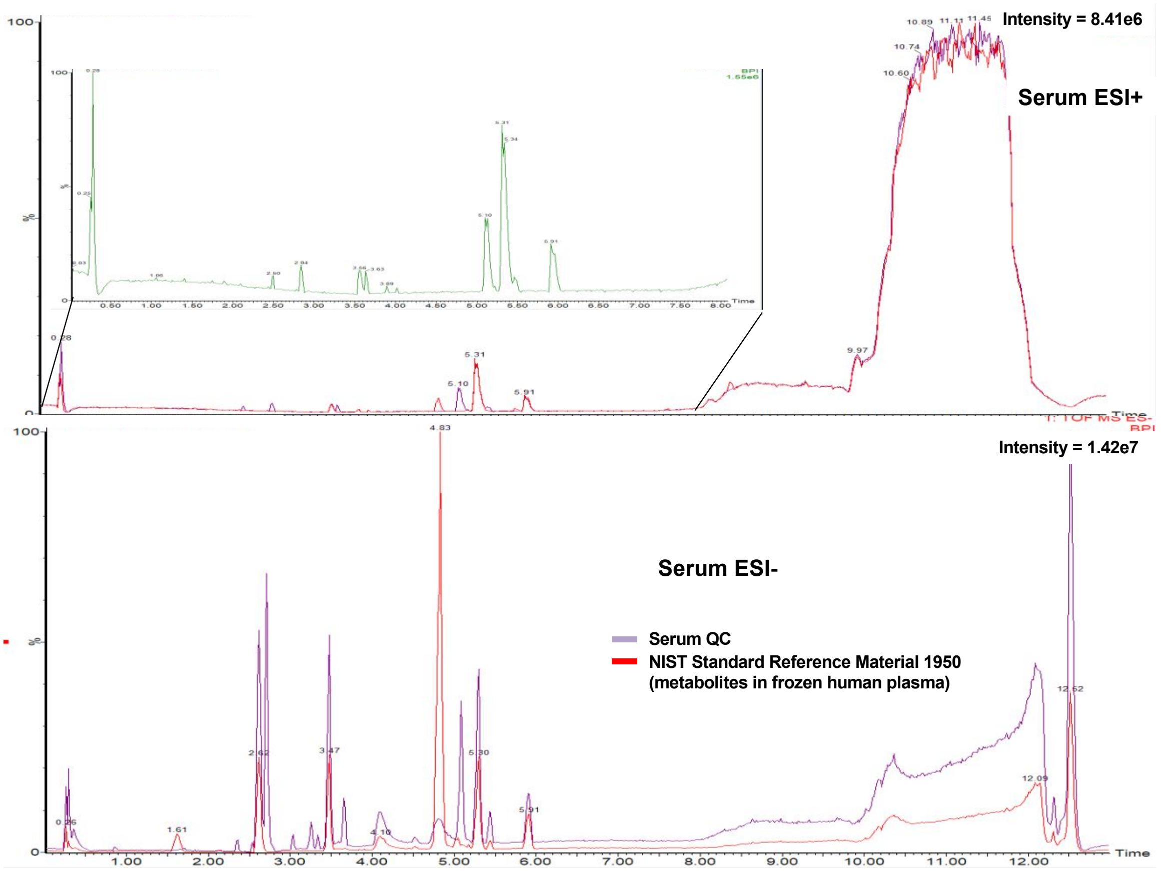

Supplemental Figure 2. Base peak chromatograms in both ESI+ and ESI- for the pooled quality control serum sample compared to NIST SRM 1950 (an insert magnifies the TIC from 0 to 8.0 min).

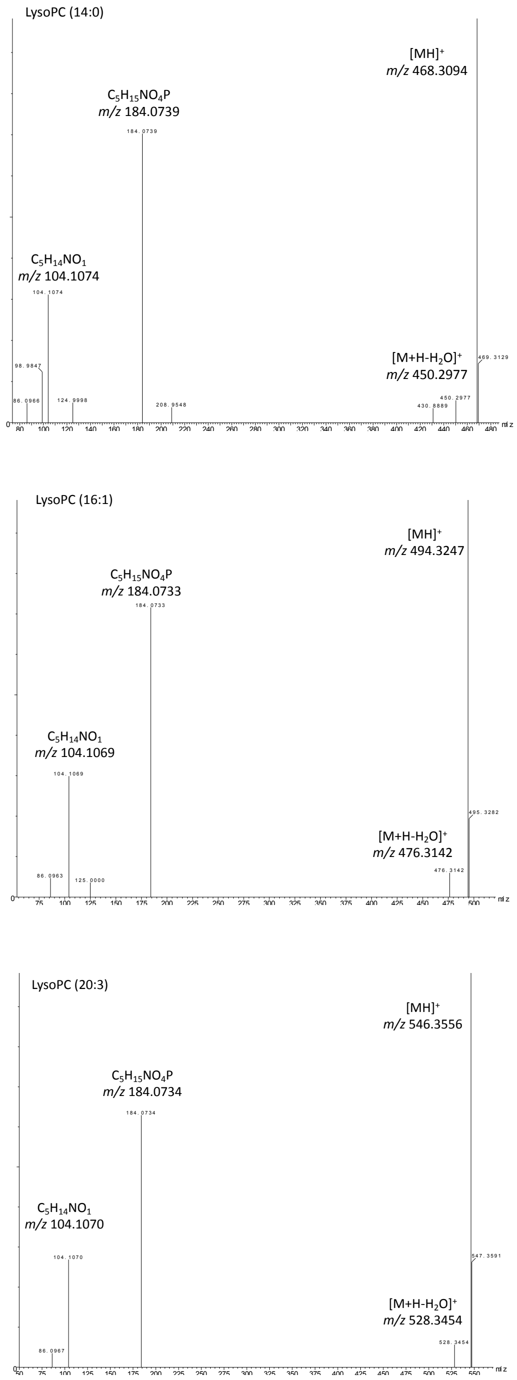

Supplemental Figure 3. Tandem MS spectra (5 – 50 V ramping collision energy) of LysoPC (14:0), LysoPC (16:1), and LysoPC (20:3).

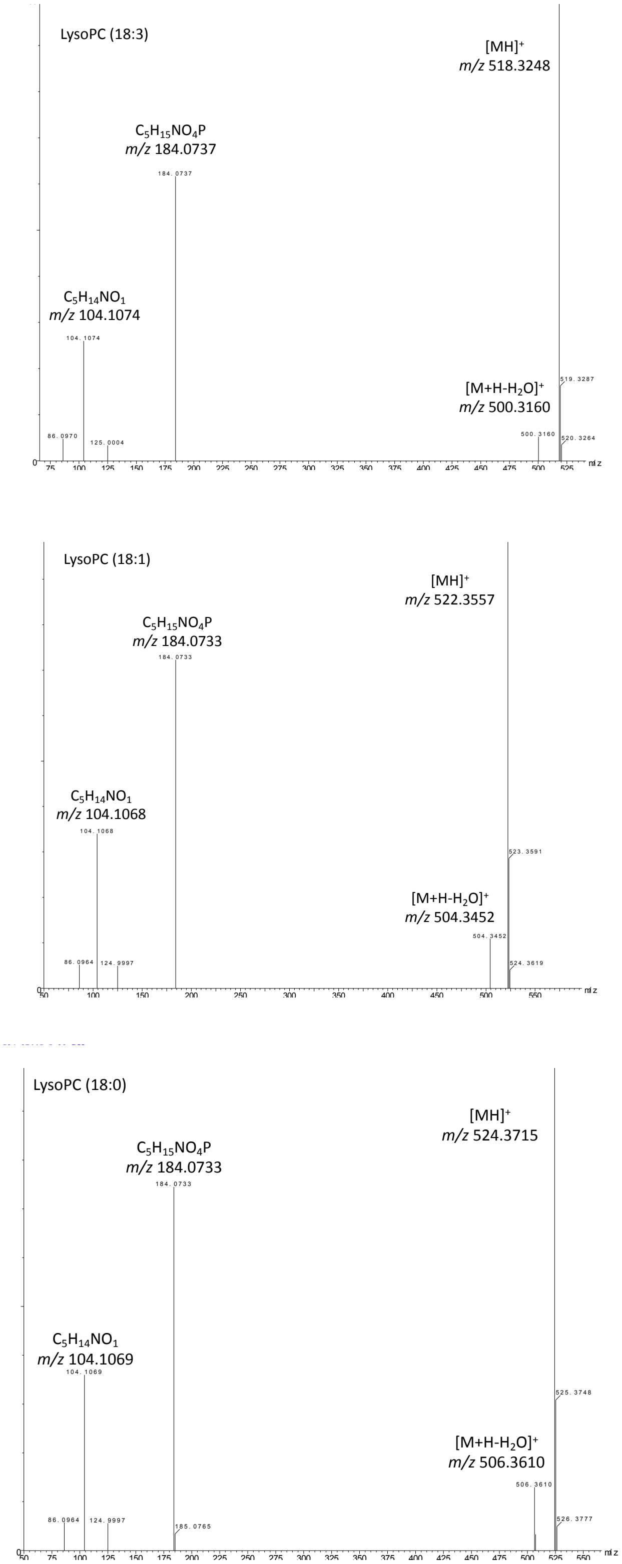

Supplemental Figure 4. Tandem MS spectra (5 – 50 V ramping collision energy) of LysoPC (18:0), LysoPC (18:1), and LysoPC (18:3).

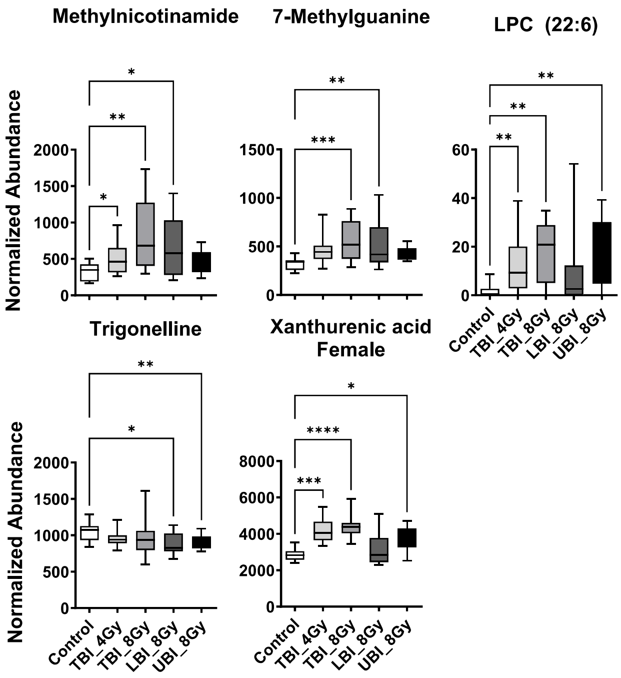

Supplemental Figure 5. Additional urinary markers that were significantly perturbed following IR exposure in the current study. Both methylnicotinamide and 7-methylguanine are promising candidates for biomarker development. However, LPC (22:6) is difficult to detect in urine, trigonelline is not repeatable and shows very low fold changes, and xanthurenic acid is specific to females.

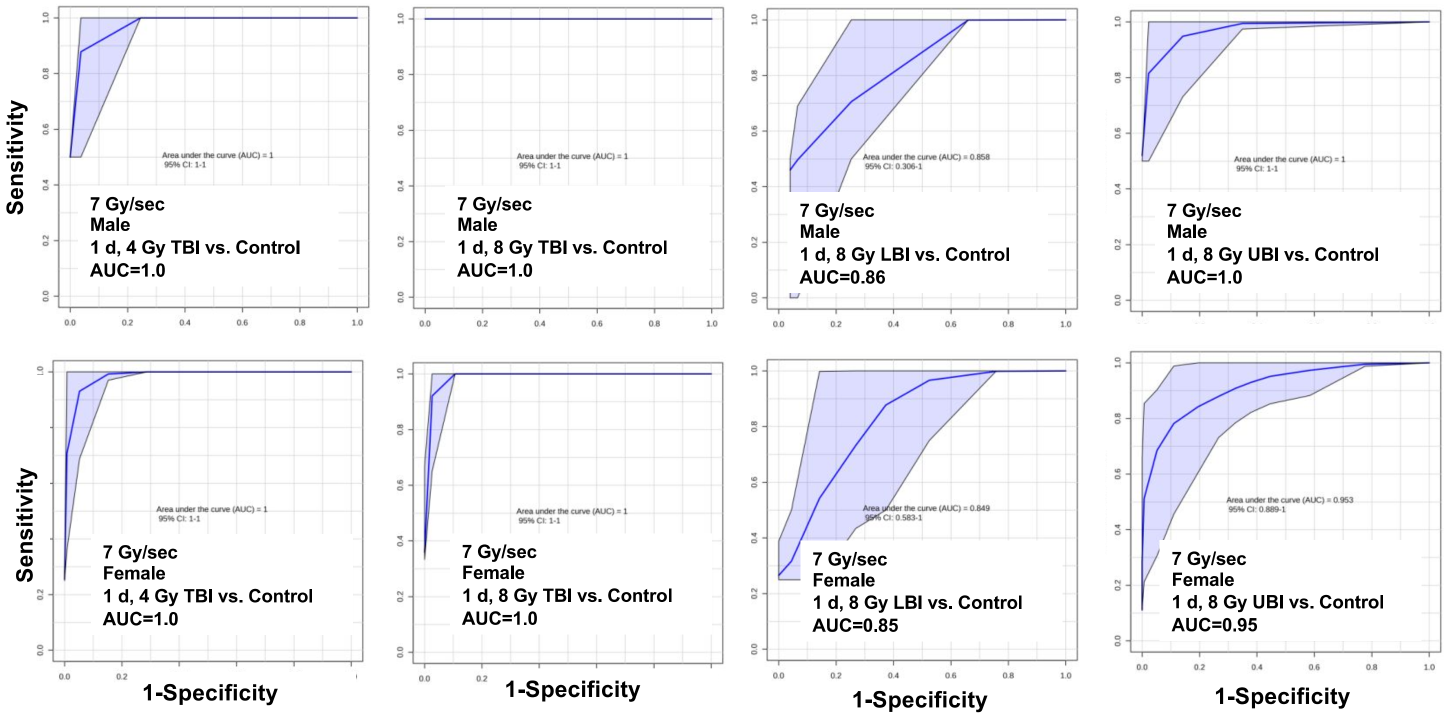

Supplemental Figure 6. Receiver operating characteristic (ROC) curves with the area under the curve (AUC) for a metabolite panel at 1 day following a TBI at either 4 or 8 Gy, a LBI at 8 Gy, or UBI at 8 Gy. The panel (N6,N6,N6-trimethyllysine [TML], carnitine, propionylcarnitine, Hex-V-I, creatine, and taurine) with males (above) and females (below) separated out shows that this panel gives excellent (AUC > 0.9) to very good (AUC > 0.8) sensitivity and specificity for partial body shielding and sex.

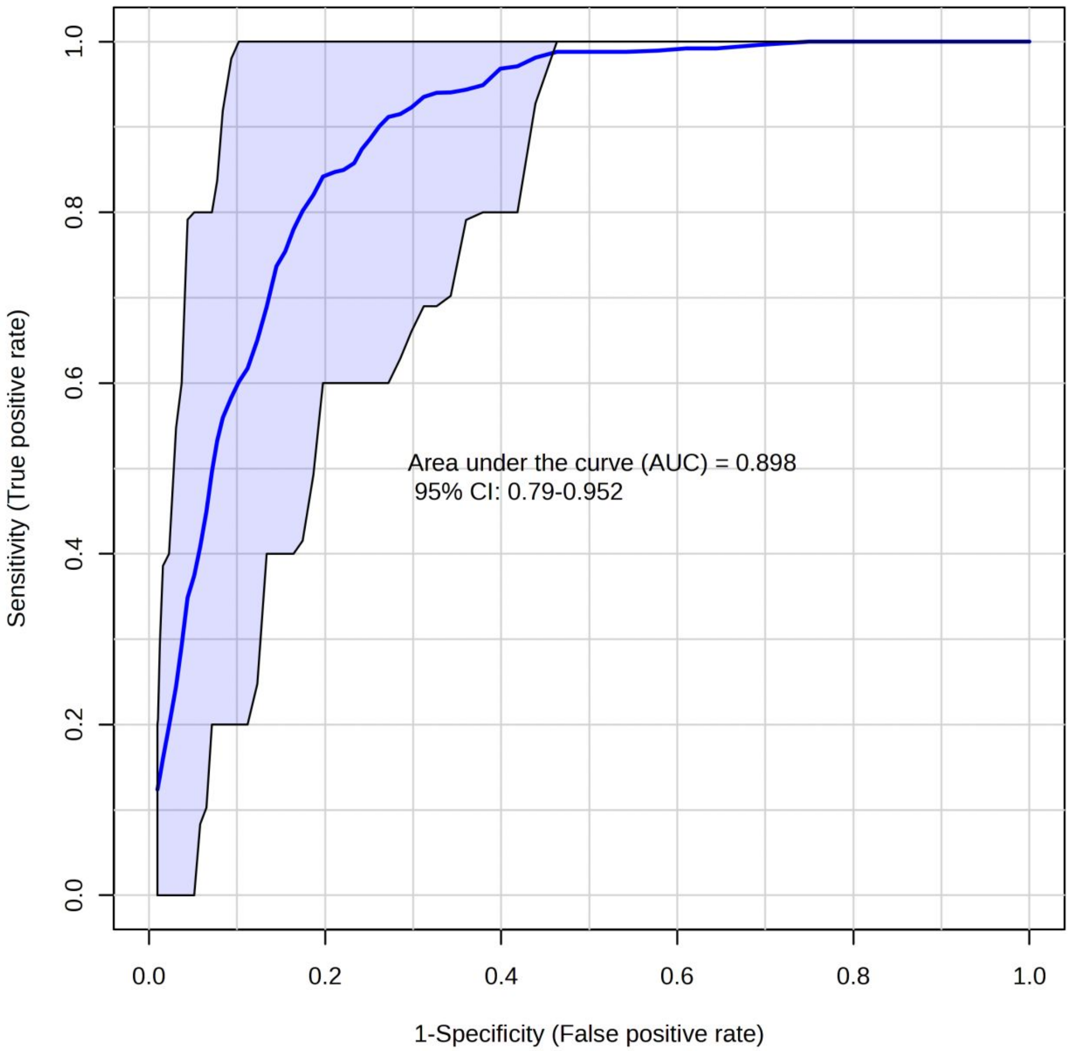

Supplemental Figure 7. Receiver operating characteristic (ROC) curves with the area under the curve (AUC) for a metabolite panel at 1 day following a TBI at 8 Gy compared to the combined sham irradiation, TBI at 4 Gy, LBI at 8 Gy, and UBI at 8 Gy groups. The panel (N6,N6,N6-trimethyllysine [TML], carnitine, propionylcarnitine, Hex-V-I, creatine, and taurine) with males (above) and females (below) separated out shows that this panel gives excellent (AUC > 0.9) sensitivity and specificity for individuals receiving higher dose TBI needing immediate medical attention compared to individuals that do not need medical care (sham) or different treatment (significant bone marrow shielding).

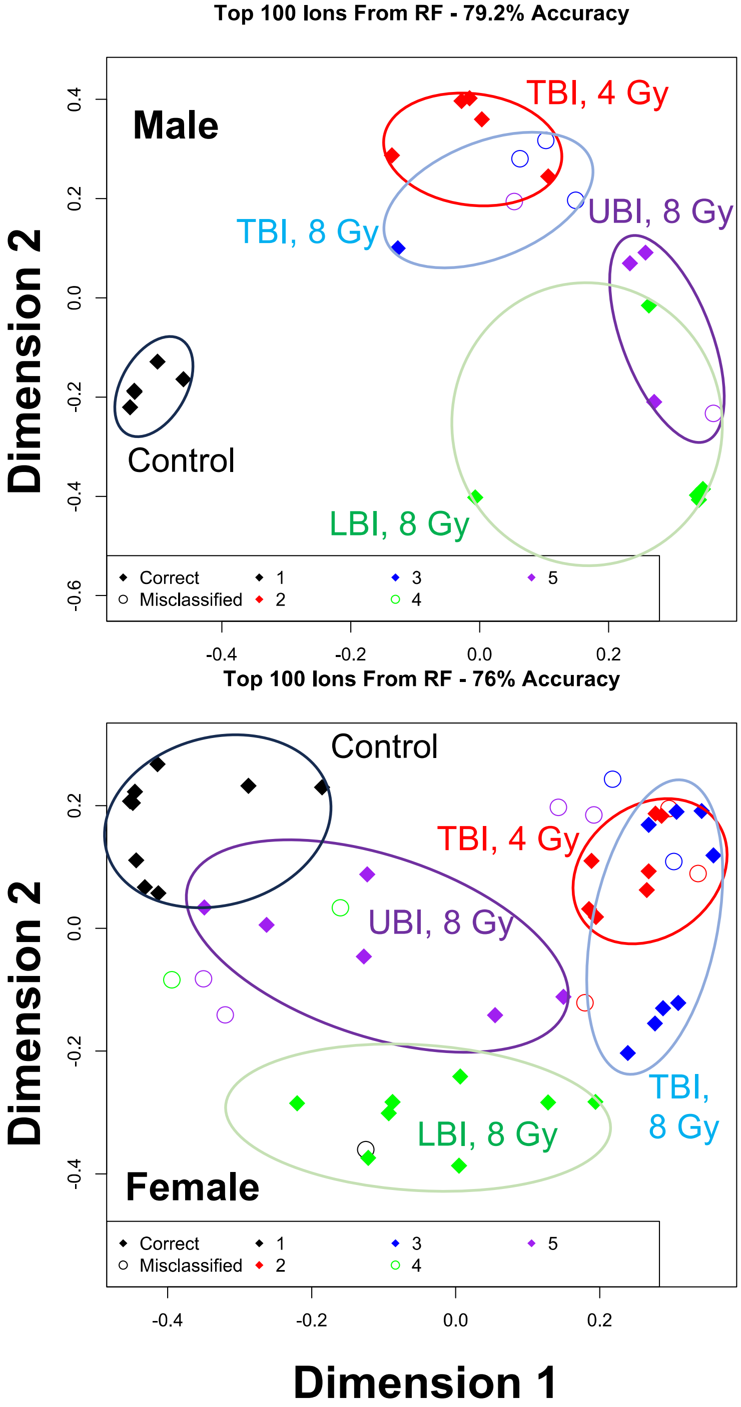

Supplemental Figure 8. Multidimensional scaling (MDS) plots were generated to visualize the combined data matrix. Compared to urine, there is a more distinct grouping between TBI and PBI groups for both males and females; however, the male PBI individuals grouped away from the control group more distinctly than females.
